# Climate-related health threats in Tanzania: a multilevel analysis of key policies, strategies, and community response

**DOI:** 10.1093/heapol/czag066

**Published:** 2026-05-14

**Authors:** Aloyce Paul Urassa, Marceline Francis Finda, Fredros Oketch Okumu, Katharina Kreppel

**Affiliations:** School of Life Sciences and Bioengineering, The Nelson Mandela African Institution of Science and Technology, P.O. BOX 447, Arusha, Tanzania, United Republic of Tanzania; Environmental Health and Ecological Sciences Department, Ifakara Health Institute, P.O. BOX 53, Ifakara, Tanzania, United Republic of Tanzania; Amplify Health and Development in Africa (AHDA), P.O. Box 8612, Moshi, Tanzania, United Republic of Tanzania; Environmental Health and Ecological Sciences Department, Ifakara Health Institute, P.O. BOX 53, Ifakara, Tanzania, United Republic of Tanzania; African Conversations Initiative, P.O. BOX 19, Ifakara, Tanzania, United Republic of Tanzania; School of Life Sciences and Bioengineering, The Nelson Mandela African Institution of Science and Technology, P.O. BOX 447, Arusha, Tanzania, United Republic of Tanzania; Environmental Health and Ecological Sciences Department, Ifakara Health Institute, P.O. BOX 53, Ifakara, Tanzania, United Republic of Tanzania; African Conversations Initiative, P.O. BOX 19, Ifakara, Tanzania, United Republic of Tanzania; School of Biodiversity, One Health and Veterinary Medicine, University of Glasgow, Glasgow G12 8QQ, Scotland; School of Life Sciences and Bioengineering, The Nelson Mandela African Institution of Science and Technology, P.O. BOX 447, Arusha, Tanzania, United Republic of Tanzania; Department of Public Health, Institute of Tropical Medicine, Nationalestraat 155, Antwerp 2000, Belgium

**Keywords:** climate change, health systems, community engagement, health policy, preparedness

## Abstract

Tanzania faces increasing climate-sensitive health threats, including vector-borne diseases, waterborne infections, and malnutrition. Effective preparedness at both national and community levels is critical for health system resilience and livelihood security. This study applied a multilevel mixed-methods approach to assess Tanzania’s policy structures and community-level experiences related to climate-health preparedness. Between January 2024 and May 2025, we conducted a concurrent mixed-methods study. At national level, we reviewed climate-health policy documents, mapped 30 key stakeholders, and conducted 15 semistructured interviews with representatives from government ministries, research institutes, development partners, and nongovernment organizations. At community level, we surveyed 388 adults and conducted eight focus group discussions in four councils in southern Tanzania. Data were analysed and triangulated across all sources. Tanzania has developed several climate-health policies and community initiatives. However, gaps remain in cross-sectoral coordination, financing, and policy implementation. National stakeholders cited challenges in translating strategies into community-level action. Among community respondents, 77% acknowledged climate change and 97% reported exposure to hazards such as floods, drought, or extreme heat. Health impacts included malaria surges, diarrhoeal disease, and food scarcity. While 73.7% had received some government assistance, access to reliable health and climate information was limited. Households relied mainly on personal observations and informal networks. Communities and institutions jointly emphasized four priorities: strengthened risk communication, climate-smart agriculture, resilient health facilities, and inclusive early warning systems. Strengthening multilevel governance, financing mechanisms, and community-driven adaptation planning is essential to improve Tanzania’s preparedness for climate-related health threats.

Key messages

- Climate change is already increasing health risks in Tanzania, including impact on vector-borne diseases such as malaria and neglected Tropical Diseases, outbreak of water and food-borne diseases such as cholera, increased malnutrition, heat stress, and gender disparities, among others.
- Although Tanzania has relevant climate and health policies, implementation remains weak at local level because financing is fragmented, coordination is inconsistent, and community-level interventions often lack clear climate-health budget lines.
- Preparedness is further limited by weak integration of climate and health data, reducing early warning, timely response, and locally relevant risk communication.
- Strengthening climate-resilient health systems requires coordinated domestic financing, routine climate-health analytics, stronger community-level implementation, and inclusive engagement of communities, women, and youth. Such reforms can improve preparedness and health outcomes while protecting development gains and reducing future emergency response costs.

## Introduction

Climate change is increasingly recognized as a major global health threat in the 21st century, with far-reaching consequences for the world’s poorest populations (Costello et al. 2009, Romanello et al. 2024, WHO 2024a, 2024b). Rising global temperatures, altered precipitation patterns, and the increased frequency of extreme weather events such as floods, droughts, and heatwaves are contributing to a growing burden of climate-sensitive diseases. The World Health Organization (WHO) estimates that between 2030 and 2050, climate change could cause an additional 250 000 deaths annually from malnutrition, malaria, diarrhoeal diseases, and heat stress alongside annual economic losses of US$2–4 billion (WHO 2024a, 2024b). These impacts are distributed unevenly. Sub-Saharan Africa faces a confluence of high baseline disease burdens, livelihoods tightly coupled to climate-sensitive agriculture and fisheries, and limited adaptive capacity within overstretched health systems (Filho et al. 2023, Marazziti et al. 2021, Opoku et al. 2021).

In Tanzania, livelihoods and economic activities are heavily dependent on climate-sensitive sectors, particularly agriculture (URT 2021a). Using the Haines and Ebi (2019) framework as a guide, Tanzania’s Climate and Health Vulnerability Assessment identified multiple climate-related health risks, including undernutrition, vector-borne and waterborne diseases, heat-related conditions, air quality impacts, injuries, and mental health effects (World Bank 2023). The Intergovernmental Panel on Climate Change (IPCC) also identified more than 20 African countries, including Tanzania, as high-risk regions for climate-related health disruptions (IPCC 2022, IPCC 2023). In many of these settings, public health infrastructures remain under-resourced, unevenly decentralized, and poorly equipped to anticipate or absorb climate shocks (Shumake-Guillemot et al. 2015). Consequently, preventative and curative capacities are overstretched, leading to increased morbidity and mortality, especially among vulnerable populations such as children, pregnant women, and the elderly.

Over the past two decades, Tanzania has made substantial public health gains. Malaria mortality has declined by >60% since 2000, driven by widespread use of insecticide-treated nets, improved case management, and strengthened health systems. Nutritional outcomes have also improved markedly. The prevalence of childhood stunting declined from ∼44% in the early 2000s to 30% in 2022, reflecting improved child health, nutrition interventions, and socioeconomic development (TDHS-MIS 2022). Regarding water access, the proportion of the population with access to at least basic drinking water services has increased significantly, rising from 28% in 2000 to 72.43% in 2022, indicating considerable gains in water infrastructure and service delivery.

However, these public health achievements face growing risks to a perfect storm of challenges such as financing, biological threats, political dynamics, rapid population growth, and climate change crises. Rising temperatures, shifting rainfall patterns, and extreme weather events pose direct and indirect threats to health systems, disease dynamics, and broader determinants of health such as food and water security. Addressing these challenges requires not only sustaining health gains but also integrating climate resilience into public health strategies at multiple levels.

Malaria is spreading into previously cool highland districts now suitable for vectors (Ryan et al. 2020), coastal and lowland zones are experiencing more frequent destructive floods, prolonged dry spells are becoming the norm, and growing seasons are shifting (Kreppel et al. 2019, Ojija et al. 2017). A study by Luhunga et al. (2018) projected a reduction in rainfall of 0.5–1 mm/day by the late 21st century under both Representative Concentration Pathway (RCP) 4.5 and RCP 8.5 scenarios, coupled with rising temperature extremes. These biophysical shifts threaten food security, safe water availability, and the continuity of health services, especially in rural areas where livelihoods hinge on subsistence farming, fishing, and pastoralism (Fadeyi and Maresova 2020, Seneviratne et al. 2012).

Currently, >70% of natural disasters in Tanzania are climate-related, including floods, droughts, landslides, heatwaves, cyclones, and wildfires (World Bank 2023, Mramba and Mapunda 2024, URT 2021b). In response, the government has ratified major international frameworks, including the UN Framework Convention on Climate Change, the Kyoto Protocol, the Paris Agreement (UNFCCC 2018), and recently endorsed the Climate and Health Declaration at the 28th Conference of Parties held in Dubai (COP28 2023).

Domestically, important climate-related policies such as the National Adaptation Programme of Action (2006), the National Climate Change Strategy (2012), and the first Health National Adaptation Plan (HNAP 2018–2023, now being updated to 2030) have also been developed (HNAP 2018, URT 2021b). These policies identify priority risks (such as malaria, undernutrition, noncommunicable diseases, cholera, and climate-induced disasters) and outline adaptation measures to strengthen health-system resilience. In addition, training programmes, health communication strategies, and stakeholder coordination platforms such as the National Framework for Climate Services have been established to enhance national preparedness (URT 2018).

Despite this policy progress, significant implementation gaps persist. Weak intersectoral coordination, limited financing for implementation, and inadequate integration of climate data into health decision-making are hindering progress (Shemsanga et al. 2010). There is also a lack of systematic engagement with communities whose lived experiences can offer vital insights into the effectiveness of policy responses (Bianco et al. 2024, Watts et al. 2015). While formal institutions recognize the importance of building climate-resilient health systems, these efforts often fail to reflect the practical realities of local populations and the knowledge who face daily health risks linked to climate change (Kahimba et al. 2015, Kitogo and Lovett 2023). Building truly climate-resilient health systems demands an iterative, people-centred approach among policymakers, health professionals, local leaders, and at-risk community members (Lugten and Hariharan 2022).

This study evaluates Tanzania’s climate-health preparedness through a multilevel mixed-methods approach that integrates national policy analysis and stakeholder perspectives with community-level evidence that captured rural households’ perceptions and lived experiences from the climate-sensitive Kilombero Valley. Specifically, it aims to (i) assess the coherence and implementation of climate and health-related policies, (ii) document community vulnerabilities and coping capacities, and (iii) identify leverage points for strengthening preparedness in resource-limited settings. By integrating top-down and bottom-up perspectives, we identify key strengths and gaps that must be addressed to support the development of inclusive, people-centred adaptation strategies in Tanzania and comparable contexts. Unlike several studies that focused either on policy or community impacts in isolation, this research provides a comprehensive, systems-level perspective that captures both institutional planning and lived experiences in a high-risk ecological zone. By focusing on the Kilombero Valley, one of the climate-sensitive areas, this study also introduces new empirical evidence on climate-linked health vulnerabilities and local adaptation needs, offering a baseline for tracking future progress and informing cross-sectoral policy responses.

## Methods

### Study design and settings

We applied a mixed-methods design to obtain complementary macro- and micro-level insights on Tanzania’s climate-health preparedness. Quantitative and qualitative data strands were collected concurrently between January 2024 and May 2025 and integrated during analysis. The study was structured around two levels of analysis, focusing on national stakeholders and local communities. The combined approach enabled triangulation of data across different sources, enhancing the reliability and comprehensiveness of the findings.

At national level, we engaged key institutions responsible for policy formulation, research, implementation, or external support on climate and health. These included government ministries, research institutions, nongovernmental organizations (NGOs), and international development partners engaged in climate change and health initiatives. This national-scale evidence was essential for assessing the strategic alignment, financing, and cross-sector coordination of climate-health preparedness in Tanzania.

**Figure 1.**
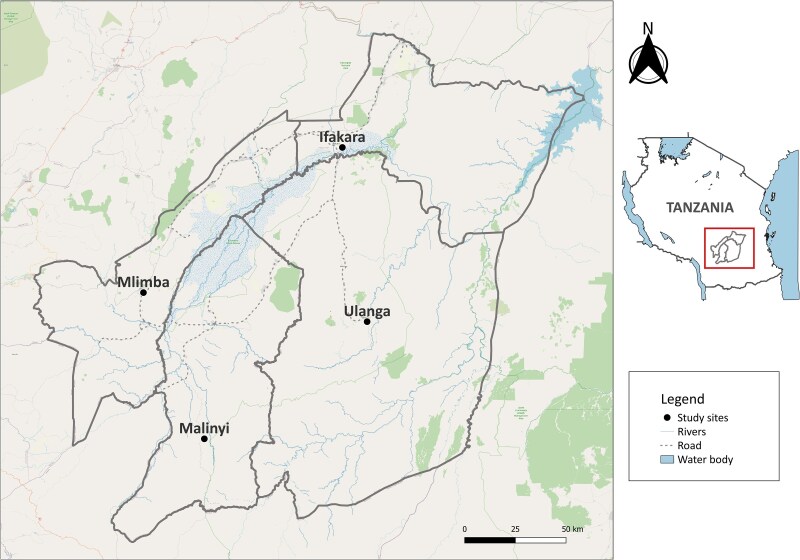
Map of Tanzania and Kilombero Valley showing the study area.

At community level, we focused on rural and urban communities in the Kilombero Valley communities including Mlimba, Malinyi, and Ifakara Town of the Morogoro Region (Fig. 1). This area is ecologically sensitive, historically endemic to malaria, and highly dependent on rain-fed agriculture, making it particularly vulnerable to climate-related health risks. The area experiences perennial *Plasmodium falciparum* transmission with significant local heterogeneity (Mukabana et al. 2025). The Kilombero Valley has experienced recurrent floods and droughts over the past decade, creating a fragile livelihood environment for communities that largely depend on rain-fed rice and maize cultivation, fishing, and livestock (Augustino et al. 2013, Fwaya et al. 2025, Johansson and Abdi 2020, Msofe et al. 2019). These attributes create a ‘hotspot’ where climate variability, disease burden, and livelihood vulnerability intersect, providing a rigorous test case for preparedness interventions (Balama et al. 2016).

### Data sources and sampling

Four main data collection strategies were employed and triangulated as follows: First, a desk review was done to examine national policy documents, strategic plans, grey literature, and international agreements relevant to climate change and health. Sources included official government publications, reports from international organizations, and development partners such as United Nations, WHO, World Bank, IOM, UNICEF, UNFCCC and UNDP. The review focused on identifying milestones, strategic priorities, and implementation gaps within Tanzania’s climate-health response framework from 1992 to 2024. Search terms were carefully selected using Boolean operators and MeSH terms where appropriate. The core search keywords included: ‘climate change’, ‘health impacts’, ‘malaria and floods’, ‘drought and nutrition’, ‘Tanzania’, ‘Kilombero Valley’, ‘community resilience’, ‘climate-health policy Tanzania’, ‘adaptation strategies’, and ‘climate variability’. Searches were refined using combinations such as: ‘climate change’ AND ‘health threats’ AND ‘Tanzania’, ‘floods’ AND ‘malaria’ AND ‘Kilombero’, ‘drought’ AND ‘nutrition insecurity’ AND ‘resilience’, ‘climate adaptation’ AND ‘health systems’, ‘policy analysis’ AND ‘climate-health Tanzania’. Inclusion required explicit health or climate-related content or budget lines, with priority given to publications from the last 10 years to ensure current relevancy. Grey literature, including government strategy documents, climate communication toolkits, and climate-health assessments from development partners, was reviewed for context and policy framing.

Second, 15 key informant interviews were conducted with purposively selected individuals from a premapped list of institutions involved in climate and health sectors, purposively distributed across four stakeholder blocks: (i) policy makers and regulators [Ministry of Health-Environmental Health Department and National Malaria Control Programme (NMCP)], Vice President’s Office, and Tanzania Meteorological Association for insights on policy, regulations and practice, (ii) research and academia (Muhimbili University of Health and Allied Sciences and KCMC University) to gain insights on research and academic engagements, (iii) civil society, and local NGOs (AMREF), Amplify Health and Development in Africa (AHDA), Climate Hub, HUDEFO, Call For Environmental Conservation, Climate Action Network-Tanzania and Youth Survival Organization to explore community engagement and resource mobilization, (iv) International organizations and development partners (WHO, and International Organization for Migration (IOM)) to understand donor priorities and coordination. The selection of these stakeholders emphasized information-rich roles. WHO and IOM were selected as international development partners because their active programmatic engagement in climate-sensitive health issues in Tanzania at the time of data collection; WHO leads technical support for climate-resilient health systems and disaster response, while IOM addresses health dimensions of climate-related migration and displacement. For these KIIs, semistructured interview guides were used to explore perceptions of climate-related health threats, preparedness levels, existing policy implementation, and recommendations for enhancing resilience. These were used to inform the relevant deductive codes during analysis. The interviews were conducted in English or Kiswahili, depending on participant preference, recorded with participant consent, and transcribed verbatim. Saturation was reached when no new themes emerged, which happened after the 15th interview.

Third, a total of eight focus group discussions (FGDs) were conducted across four councils, including Ifakara Town, Malinyi, Ulanga, and Mlimba. Participants were purposively selected with assistance from local leaders (village chairpersons, ward, and village executive officers), who identified potential participants, using criteria that included residence duration (minimum 2 years, with the expectation that this is a group that would have a better understanding of the community situation with regard to the topic), community engagement, and experience with local environmental and health challenges. Each FGD consisted of 6–10 participants, stratified by gender and mixed age group to ensure diversity of perspectives and capture intergenerational and gendered experiences of climate and health impacts.

A structured discussion guide was developed to guide the FGDs, covering themes such as awareness and understanding of climate change, perceived and observed health impacts, local adaptation strategies, and access to health and climate information. Prior to the discussions, participants were oriented on key terms and concepts to mitigate the risk of conceptual ambiguity. For instance, the term ‘mabadiliko ya tabianchi’ was used to refer to climate change, ‘mabadiliko ya hali ya hewa’ to weather change, and ‘mabadiliko ya misimu’ to seasonal variation. Facilitators carefully explained these distinctions using local examples (e.g. long-term rainfall pattern shifts vs. abrupt unseasonal rains) to ensure conceptual clarity.

Data from FGDs were collected in Kiswahili by trained facilitators and note takers. All discussions were audio-recorded, transcribed verbatim, and translated into English for analysis.

Lastly, a structured questionnaire was administered to a random sample of 388 adults across the four study councils, all aged 18 years or older. Sample size estimation was estimated using quantitative sample size (*n*) calculation (Pourhoseingholi et al., 2013):


$$n=Z^{2}x\frac{\;p(1-p)}{e^{2}}$$

where; Z = z score at 95% CI (1.96); e = marginal error at 95% CI = 0.05; p = Proportion of the population =50%


$$n=1.96^{2}x\frac{0.5(1-0.5)}{0.05^{2}}$$

This yielded a minimum required sample of 385 respondents. A total of 388 completed questionnaires were obtained, slightly exceeding the minimum to account for potential nonresponses or incomplete records. Enumeration areas were sampled proportional to population size, and households were selected using a random walk approach. The questionnaire captured sociodemographics, climate change awareness, self-reported health impacts, disaster exposure, and sources of information. Trained fieldworkers conducted face-to-face interviews with the head of household or, in their absence, the most senior adult member present at the time of the visit, to ensure consistent and complete responses.

### Data management and analysis

The quantitative data from the household surveys was analysed using SPSS version 26 (IBM Corp 2019). Weighted descriptive statistics (means, proportions) and χ^2^ tests were generated to compare awareness and impact perceptions across councils. All analyses were performed at a 95% confidence interval, with *P* < 0.05 considered statistically significant.

All audio-recorded interviews and focus group discussions were transcribed verbatim to ensure familiarity with the data. A mind-mapping technique was used to reflect on and organize the information in relation to the study objectives, serving as a foundation for analysis (Fearnley 2022). Transcripts were imported into NVivo 12 Plus, and insights from the mind map guided interpretation (Scopus Database 2023).

To ensure the quality of the qualitative data analysis, a hybrid inductive-deductive coding approach was used to align the study objectives and key policy domains. Inductive codes emerged from participants’ responses, while deductive codes were developed *a priori* based on the study’s conceptual framework and relevant literature. The analysis focused on four main domains: climate exposure, health impacts, adaptation strategies, and policy awareness and implementation. To ensure validity, two individuals independently coded the transcripts and compared their coding; any discrepancies were discussed and resolved by reaching an agreement.

### Ethical considerations

Ethical approval was obtained from the Institutional Review Board of the author’s institute with reference number IHI/IRB/No:28-2024. Additionally, the key stakeholders and local authorities granted permission, and all participants provided written informed consent. Identifiers were removed during analysis and reporting.

## Results

### Characteristics of study participants

A total of 388 community members completed the household survey (Table 1), 64 community members participated in eight Focus Group Discussions (Table 2), and 15 stakeholders participated in Key Informant Interviews (KII). The survey participants (*N* = 388) were distributed by gender with 220 male (56.7%) and 168 female (43.3%), with a mean age of 42.6 ± 13.5 years. From the survey, 255 participants (65.7%) had completed only primary schooling, and 246 participants (62.1%) relied mainly on rain-fed farming as their main source of income, the remainder being small traders, teachers, or drivers. Geographic coverage of the survey mirrored actual population densities in the study area (Ministry of Finance and Planning et al. 2022): 177 survey participants (46%) lived in Ifakara Town Council, 130 survey participants (34%) in Ulanga, and the rest in Mlimba and Malinyi (Fig. 1).

**Table 1:** Demographic information for survey participants from Ulanga, Malinyi, Mlimba, and Ifakara town, Tanzania (*N* = 388).

Category	Variable	Number of participants (%)
**Sex**	Male	220 (56.7)
	Female	168(43.3)
**Age group**	18–35 years	147 (37.89)
	36–59 years	187 (48.2)
	60 and above	54 (13.92)
	Mean age = 42.64 SD = 13.51	
**Education level**	No formal education	15 (3.87)
	Primary school	255 (65.72)
	Secondary school	103 (26.55)
	Diploma/certificate (≥1 year postsecondary)	10 (2.58
	University degree (≥3 years postsecondary)	5 (1.29)
**Type of work or employment (main activity)**	Agriculture/farming	246(63.4)
	Entrepreneurship/business	117 (30.15)
	Teaching	10 (2.58)
	Drivers	05 (1.29)
	Homecare	07 (1.80)
	Pastors	03 (0.08)
**Location**	Ifakara town	177 (45.62)
	Ulanga district council	130 (33.51)
	Mlimba district council	41 (10.57)
	Malinyi district council	40 (10.31)

**Table 2:** Characteristics of the Focus Group Discussions Participants from Ifakara Town, Ulanga, Malinyi and Mlimba communities in Morogoro, Tanzania (*N* = 64).

Category	Variable	*n* (%)
**Sex**	Male	32 (50.0%)
	Female	32 (50.0%)
**Age (years)**	Mean ± SD	41.1 ± 11.7
	Range	21–77
**Age group**	18–35 years	24 (37.5%)
	36–59 years	35 (54.7%)
	≥60 years	5 (7.8%)
**District/Council**	Ifakara Mjini	16 (25.0%)
	Ulanga	16 (25.0%)
	Mlimba	16 (25.0%)
	Malinyi	16 (25.0%)
**Main economic activity**	Agriculture	47 (73.4%)
	Entrepreneurship	10 (15.6%)
	Teaching	5 (7.8%)
	Entrepreneurship/business	2 (3.1%)
**Time lived in the area (years)**	Mean ± SD	28.9 ± 16.6
	Range	3–65

### Evolution of the climate-health policy landscape (1992–2024): results of the desk review

**Figure 2.**
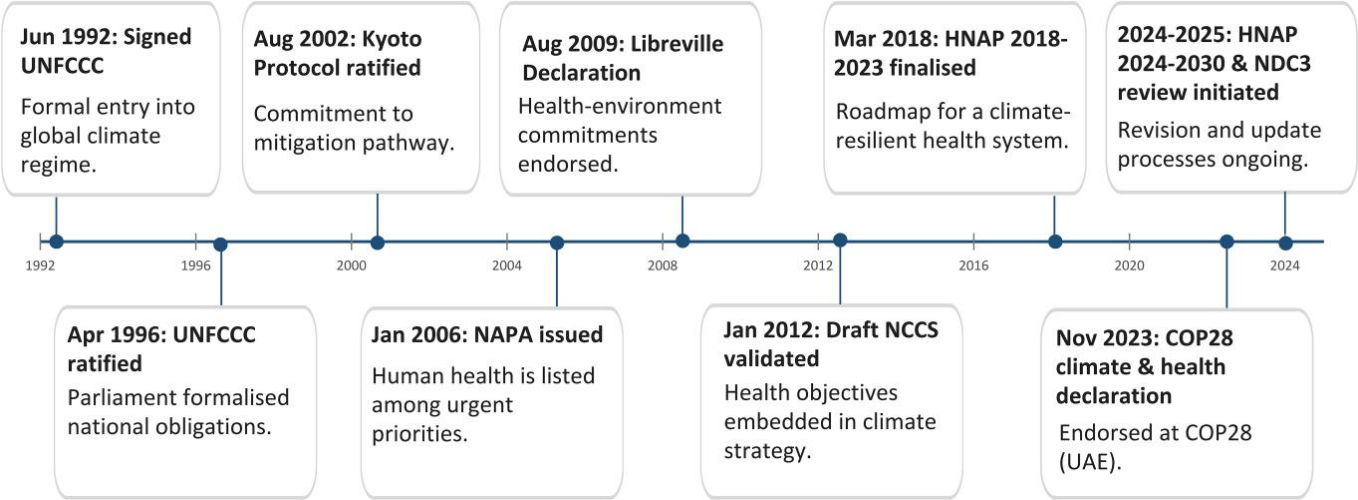
Evolution of climate-related health policies in Tanzania (1992–2025).

Overall, Tanzania’s trajectory shows clear policy expansion and growing multisector collaboration on key aspects of climate and health (Fig. 2; Supplementary Table S1), though there are also persistent gaps in funding and local execution. The desk review revealed a steady but uneven maturation of Tanzania’s climate-health policy architecture since signing the United Nations Framework Convention on Climate Change (UNFCCC) in 1992 at the Earth Summit in Rio de Janeiro. The convention provided the overarching legal framework for international cooperation to stabilize greenhouse-gas concentrations and underpinned subsequent agreements such as the Kyoto Protocol and the Paris Agreement. In Tanzania, early policy engagements centred on these high-level commitments, notably the UNFCCC (1992, ratified 1996) and Kyoto Protocol (2002), followed by the first National Adaptation Programme of Action in 2006, which formally listed human-health priorities. Momentum accelerated after 2009 with the Libreville Declaration and culminated in the 2012 National Climate Change Strategy that embedded health objectives across sectors. This was also Tanzania’s first national strategy on climate change and related impacts.

From 2014 onward, Tanzania entered an ‘operationalization phase’, which included the establishment of a National Framework for Climate Services and roll-out of Global Framework for Climate Services (GFCS) pilot projects to improve production, accessibility, and application of climate information. In Tanzania, these GFCS pilot projects were aimed at promoting collaboration between climate scientists and user sectors such as health, agriculture, water, and disaster risk reduction. This enhanced decision-makers ‘ability to receive tailored, timely climate services for planning and risk management. The operationalization phase also included malaria-specific climate data briefings and capacity-building workshops for meteorologists and health officers.

The publication of the Climate Change and Health Communication Strategy (2016) and the Health National Adaptation Plan (HNAP 2018–2023) marked the first comprehensive attempt to forge a climate-resilient health system. However, implementation reviews have since highlighted financing gaps and limited subnational uptake of this plan. A scheduled HNAP update (2024–2030) and the third Nationally Determined Contribution are expected to provide opportunities to address these shortcomings. Figure 2 provides a visual synthesis of key milestones identified in the desk review. A concise summary of the milestones is provided in Supplementary Table S1.

### Stakeholders mapping

Stakeholder mapping identified a multilevel governance ecosystem spanning individuals and communities, subnational planning structures, national ministries and agencies, and international partners. At community level, households, farmers, women and youth groups, and community health workers (CHWs) were positioned as primary risk-bearers and first responders, with high stakes (interest) but relatively limited formal influence over resource allocation. Community leaders and frontline implementers (e.g. CHW coordinators, village leaders, teachers, and local civil society/youth-led organizations) were described as critical intermediaries for risk communication, behaviour change, and locally feasible adaptation actions. At district and regional levels, Council Health Management Teams and sector officers (health, environment, agriculture) were identified as pivotal for translating national strategies into integrated annual plans, coordinating cross-sector implementation, and linking ecosystem and disease surveillance. At national level, the Ministry of Health (including the National Malaria Control Programme), the Vice President’s Office (Environment), Prime Minister’s Office- Regional Administration and Local Government (PMO-RALG), and the Tanzania Meteorological Authority were consistently described as central nodes for policy direction, coordination, and climate services provision. The Ministry of Finance and parliamentary committees were mapped as high-influence actors shaping fiscal space and accountability. International development partners and climate/health financing institutions (e.g. WHO, UNDP, and climate funds) were positioned as influential due to their technical assistance and financing roles, particularly for piloting and scaling climate-health initiatives.

**Figure 3.**
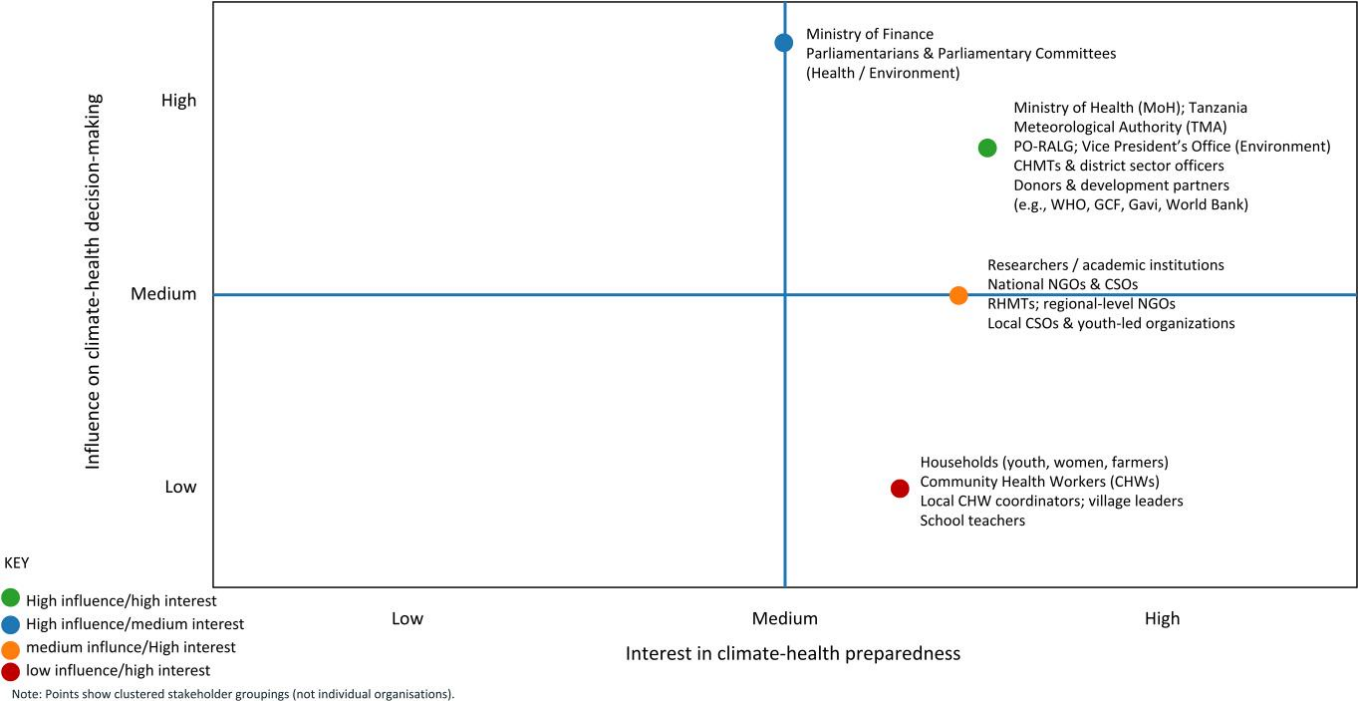
Stakeholder influence/interest matrix used in stakeholder mapping during the multilevel study in Tanzania.

Figure 3 summarizes stakeholder positioning along an influence-interest grid, derived from the stakeholder mapping and triangulated with KIIs while, Supplementary Table S2 provides further details.

The keywords used in mapping were described as follows:

**Operational level**: the governance or implementation tier at which a stakeholder primarily operates in Tanzania’s climate-health system, individual/household, community, regional (district/regional authorities), or national (ministries/central agencies).

**Role**: the stakeholder’s core function in climate-health preparedness and response, e.g. experiencing impacts and driving behaviour change (community members/CHWs), local mobilization and awareness, planning and implementing adaptation/resilience actions (CHMTs/RHMTs), or policy leadership, coordination, and technical services (Ministry of Health, Vice Presidents Office-Environment, TMA, PMO-RALG).

**Level of influence (influence/engagement level)**: a simple high/medium/low rating of how much a stakeholder can shape decisions, priorities, and resource allocation, and drive coordination or implementation in climate-health work (with ‘high’ typically for national policy/technical authorities and district planners; ‘medium’ for active CSOs/regional implementers; ‘low’ for groups mainly affected or engaged locally without formal decision power).

### Stakeholder awareness and perceptions of climate change

Overall, 299 (of the survey participants (77%) reported knowing about climate change although follow-up probing showed partial misconceptions. When asked to define the term, 169 participants (43.6%) supplied a basic but essentially correct description, i.e. ‘long-term changes in temperature or rainfall’, whereas 74 participants (19.1%) equated it with short-term weather variability and 9.3% were unsure. Moreover, 163 participants (42.0%) stated there was ‘no difference’ between weather and climate, underscoring a communication gap already flagged in KIIs. Common references from open-ended questions included quotes such as: ‘The weather has changed,’ ‘the rain no longer falls like it used to,’ ‘rainfall has decreased,’ ‘the sun is very intense.’

From the survey, 234 participants (60.3%) of the participants learned about climate mainly from television followed by radio (Table 3). Only 23 participants (6%) during the survey had received forecast information directly from the Tanzania Meteorological Authority (TMA), and some 51 participants (13%) relied on family or friends. Open-ended answers on any other sources revealed that 74 participants (19.07%) of survey participants, alongside the main sources they still depended on personal observation, e.g. ‘watching the sky’ as a way to decide on their farming decisions.

**Table 3:** Common sources of information concerning climate change reported by survey participants in Ulanga, Mlimba, Malinyi and Ifakara town, Tanzania.

Source	Frequency	Percentage (%)
**Television**	234	60.3
**Radio**	156	40.2
**Family/friends**	51	13.1
**Local government meetings**	28	7.2
**Tanzania meteorological authority**	23	5.9
**Magazines**	3	0.8
**Others (mobile phones, social media platforms and community announcements, books, religious teachings)**	74	19.1

#### Linking climate change to health outcomes

Nearly all survey participants had experienced at least one climate-linked hazard in the previous 3 years, with 377 participants (97.2%) reporting such exposure (Table 4). The most frequently reported hazards were drought reported by 253 participants (65.2%), delayed rains by 211participants (54.4%), and extreme heat reported by185 participants (47.7%), followed by other impacts such as crop failure, disease outbreaks, and hunger reported by154 participants (39.7%), and floods reported by121participants (31.2%).Open-ended feedback from survey comments echoed FGD narratives such as: ‘We no longer know when to plant’, or ‘Because of the drought, we didn’t harvest anything’. Participants commonly linked floods with malaria surges and noted that unsafe water after heavy rains ‘brings stomach illnesses’.

**Table 4:** Climate-related disasters faced by participants of questionnaire survey from Ulanga, Mlimba, Malinyi, and Ifakara town, in Morogoro, Tanzania.

Climate-linked hazards	Frequency (number of respondents)	Percentage (%)
**Drought**	253	65.2
**Delayed rainfall**	211	54.4
**Extreme heat**	185	47.7
**Others (crop failure, diseases outbreak, hunger, etc.)**	154	39.7
**Floods**	121	31.2
**Unpredicted rainfalls**	120	30.9
**Extreme rainfall**	66	17.0
**Storms**	35	9.0
**Wildfires**	2	0.5

Across the key informants, policy makers, development partners, researchers, and civil society, there was strong convergence that climate hazards are no longer ‘emerging’ but already disrupting health outcomes and service delivery. Informants repeatedly linked climate variability and extreme events to malaria pattern shifts, including resurgence in higher altitude areas, expansion of dengue risk in coastal and urban settings, and flooding episodes that precipitate cholera and other waterborne outbreaks, with knock-on effects on productivity and household income. While most recognized that Tanzania has developed a substantial policy architecture (e.g. national strategies, NDCs, and sectoral plans), they described preparedness as uneven, stronger on paper than in routine implementation largely because financing, coordination, and systems integration remain inconsistent.

While national preparedness structures exist… there is often a disconnect between expert knowledge and community-level needs. (Male-Local NGO)

## Stakeholders’ insights on climate-related health threats and preparedness

### Perceived current state and agency

Community survey participants expressed both a sense of responsibility and expectations of government leadership. When asked who should take the greatest role in addressing climate-related health impacts, 276 participants (71.1%) selected government and 256 participants (66.0%) selected community members (multiple response question), suggesting shared responsibility. Consistent with this, 323 participants (83.5%) agreed that community members can play a role in reducing health impacts. Perceived feasible community actions included environmental conservation reported by 203 participants (52.3%), tree planting reported by 133 participants (34.3%), protecting water sources by 120 participants (30.9%) and community education on climate and health reported by 98 participants (25.3%; see Table 5).

**Table 5:** Selected survey indicators related to perceived roles and preparedness (*N* = 388).

Indicator (survey)	Key response options	Result
**Who should take the greatest role? (multiple responses)**	Government; community members	71.1%; 66.0%
**Communities can play a role to reduce health impacts**	Yes	83.5%
**Government provides any support to help communities prepare**	Yes; No; Don’t know	73.7%; 19.6%; 6.7%
**It is possible to control harms of climate change**	Yes; No; Don’t know	75.0%; 18.8%; 6.2%

### Budget and resource allocation constraints

The climate-health financing gap was described as fragmented and insufficiently aligned across levels. A recurring concern was the existence of multiple funding channels (central treasury allocations, dedicated climate funds, and time-bound project grants) that operate in parallel, with weak integration into routine health planning and limited predictability for subnational implementation. Several stakeholders highlighted that climate risk is not consistently translated into budget lines within Council health plans, which limits the ability of districts to implement locally relevant preparedness actions (e.g. outbreak readiness, Water, Sanitation, and Hygiene (WASH) contingencies during floods, community-based vector control). The overall interpretation was that the main constraint is not a lack of ‘plans’ but the lack of sustained, domestically anchored financing and prioritization mechanisms that push climate-health actions into annual budgeting cycles.

To ensure preparedness, we need strong political will to put climate and health on their priority list during budgeting. (Male, Development Partner)

### Data and analytics gaps for early warning and targeting

Key informants consistently emphasized that the data backbone for climate-health preparedness remains underdeveloped. Researchers and technical actors pointed to limited routine integration of meteorological information with disease surveillance, which reduces the feasibility of timely, actionable early warning at district and community levels. Civil society participants added that risk communication often proceeds without sufficiently localized evidence, and that messages can be too generic to guide household or facility-level preparedness. Importantly, informants framed this not as a one-off ‘pilot gap’, but as a systems gap: insufficient routine climate-health analytics, limited disaggregation for subnational decision-making, and constrained data infrastructure (storage, access, and interoperability) that reduces the operational relevance of expert input.

Most of the policies and available data are at a very high-level lacking disaggregation that is important for response and preparedness at the sub-national level. (Female, Academic Institution)

### Communication and coordination challenges across governance levels

Communication and coordination were described as persistent bottlenecks across institutions responsible for climate, health, disaster management, and local government. Informants noted that even where formal coordination platforms exist, information does not consistently move in ways that support rapid local decision-making, and community priorities do not reliably travel back up into planning and resource allocation. Several participants stressed that communication strategies often underperform because they do not reflect local realities (language, livelihoods, seasonal risks, and trust dynamics), leading to low uptake of guidance. In practical terms, informants advocated for clearer information pathways, defined roles for frontline actors (including Community Health Workers), and more context-responsive communication products linked to locally observed risk patterns.

Clear communication and proper channelling of information give power to the people to anticipate and prepare for what may happen tomorrow. (Male, Civil Society Organization)

### Youth engagement as an underutilized capability

Youth engagement was viewed as increasingly active in climate-health advocacy and mobilization, but insufficiently integrated into formal planning and resourcing. The dominant interpretation was that youth involvement is often episodic-focused on implementation and campaigns rather than embedded in agenda-setting, budgeting, and accountability processes. Informants therefore framed ‘meaningful youth engagement’ as requiring structured pathways (leadership roles, predictable financing windows, and defined participation mechanisms) that connect youth-led initiatives to district and national climate-resilient health planning.

Youth have ideas and energy, but we are often not included in the planning stages; we need to be brought in earlier, not just for implementation. (Male, Youth-Led Organisation)

### Experienced climate variability and livelihood disruption

Across the eight FGDs, participants consistently described a perceived shift from predictable seasonal patterns to erratic rainfall and less reliable temperature cycles. This ‘loss of seasonality’ was not presented as an abstract concept, but as a practical disruption to farming calendars, household planning, and risk anticipation. Participants described variability in onset and duration of rains, alongside episodes of drought and flooding, which together undermine livelihoods and heighten vulnerability to health shocks.

We no longer know when to plant. The seasons have changed too much and the rain no longer follows a pattern. (Female Community Member)

### Health effects linked to floods, vectors, heat stress, and psychosocial strain

Participants commonly connected climate variability to increased malaria risk (including perceived mosquito increases and illness outside ‘expected’ seasons) and to waterborne disease during floods when sanitation systems fail and water sources become contaminated. In addition to infectious disease, there were references to heat-related illness and a clear narrative of psychosocial stress, especially anxiety associated with crop failure, reduced income, and the compounding costs of illness. Overall, the findings indicate that climate-related health vulnerability in these settings is multi-pathway: direct exposure (heat), ecological change (vectors), infrastructure disruption (water/sanitation), and socioeconomic stressors (food and income)

When floods come… toilets overflow, drinking water gets contaminated, and children start getting diarrhoea. (Male Community Member)

### Agriculture and food insecurity as the dominant pathway of vulnerability

Food systems emerged as a central lens through which communities understood climate risk. Participants described rainfall variability and floods as drivers of crop loss, reduced yields, and unstable household food supplies. These disruptions translated into reduced meals, lower dietary diversity, increased reliance on coping labour, and borrowing–highlighting how climate impacts cascade beyond agriculture into nutrition, household stress, and health-seeking capacity. In these communities, climate–health preparedness was therefore viewed as inseparable from agricultural resilience and food security measures (inputs, storage, and adaptive practices).

We used to eat three times, but now sometimes we only eat once. (FGD, Ulanga)

### Coping strategies and adaptation practices

Participants described pragmatic, locally driven coping strategies, including switching to early maturing crops, diversifying income through temporary migration or casual labour, using traditional remedies, and relying on faith-based coping. While these strategies demonstrate adaptive agency, they were also framed as partial and sometimes fragile responses, often insufficient against recurrent shocks and constrained by limited access to improved inputs, finance, and timely information. The overall interpretation is that communities are adapting, but largely through incremental and self-financed adjustments rather than through supported, risk-informed preparedness systems.

We are now planting early-maturing maize to reduce the risk of losing everything when rains stop suddenly. (Female Community Member)

### Priority needs for preparedness and service resilience

Participants expressed clear priorities: closer and better-equipped health facilities to manage climate-related emergencies, early warning and timely risk communication, and improved access to agricultural inputs (e.g. drought-resistant seeds) and storage infrastructure. These priorities indicate that communities conceptualize preparedness as a package of services and systems, healthcare access, information systems, and livelihood supports rather than a single intervention. They also emphasized the need for more visible and responsive government support at local levels during climate-related shocks.

No one tells us in time when something big is going to happen. By the time we know, we are already affected. (Male Community Member)

### Gendered vulnerability and unequal burden of response

Gendered patterns were prominent across FGDs and echoed by key informants. Women and girls were described as carrying disproportionate responsibility during climate-related crises due to caregiving roles, water and fuel collection burdens (which intensify during drought), and constrained access to household resources that shape health-seeking and nutrition. Participants also highlighted gendered psychosocial impacts, with women describing stress tied to feeding families, securing treatment, and managing household welfare during repeated shocks. Key informants additionally referenced emerging policy attention to climate–gender linkages, indicating an opportunity to institutionalize gender-responsive approaches in climate-resilient health planning.

Women are the ones who fetch water and firewood. During droughts, we walk longer distances and often face illness due to heat and carrying heavy loads. (Female Community Member)

#### Recommendations to strengthen preparedness

Key stakeholders articulated convergent priorities for strengthening preparedness: institutionalize cross-sector coordination mechanisms that link climate services, surveillance, and response planning from national to council levels; secure predictable financing for climate-health actions within government budgeting processes, including dedicated line items in council health plans; strengthen integrated data systems and local analytical capacity for climate-informed early warning and targeting; expand locally tailored risk communication channels that leverage Community Health Workers, schools, and other trusted community structures; and support livelihood resilience measures (notably climate-smart agriculture, water management, and sanitation improvements) that reduce downstream health risks. Community narratives additionally emphasized practical needs such as resilient health facilities, improved access during floods, and timely alerts to enable anticipatory action.

## Discussion

This multilevel study examined Tanzania’s preparedness for climate-related health challenges by integrating analysis of national policy frameworks with the lived experiences of affected communities. The findings indicate that Tanzania has established a relatively robust policy architecture for climate-resilient health systems, including early ratification of international climate agreements, the 2012 National Climate Change Strategy, and the Health National Adaptation Plan (HNAP 2018–2023). However, insights from key stakeholders and communities consistently highlighted that the translation of these frameworks into effective action at community level remains limited.

Key informants acknowledged the relevance of existing policies but identified fragmented financing and weak vertical coordination as persistent constraints. At community level in the Kilombero Valley, widespread hazard exposure coupled with limited conceptual understanding of climate change and very low direct uptake of TMA forecasts underscored a critical disconnect between national climate services and local-level utilization.

These patterns in Tanzania mirror evidence from other African settings. For example, in western Kenya, 76% of rural respondents had heard of climate change, yet conceptual misunderstandings were common, and 93% linked it to worsening health threats (Greibe Andersen et al. 2023). Studies in Malawi and Mozambique likewise document sophisticated national plans set against local information gaps and donor-dependent financing (Fisher and Oppenheimer 2021, Jørstad and Webersik 2016, González et al. 2021, Sharma et al. 2024).

The findings also support the ‘awareness–action gap’ described by Opoku et al. (2021), who noted that communities intuitively recognize changing seasons. However, structural constraints such as poverty, limited extension services, and under-resourced health posts reduce their ability to proactively adapt to these changes. The repeated linkage of drought and floods to both crop failure and malaria spikes aligns with ecological observations that altered rainfall patterns, extend *Anopheles* breeding windows, and reduce household nutritional status, compounding susceptibility to infections and nutritional deficiencies (Cochet et al. 2024, Mayala et al. 2015, Mkonda and He 2018, Mwangungulu et al. 2023).

These findings have direct and indirect implications for policy and practice, both in Tanzania and other low and middle-income countries facing similar challenges. There is a direct need for improved financing and governance. The desk review highlighted a decade of increasing multisector collaborations in Tanzania (e.g. GFCS pilot projects), while stakeholder interviews exposed parallel funding streams that are not well integrated, often insufficient and sometimes redundant in operations. This suggests the need for establishing an earmarked climate-health budget line, e.g. within the medium-term expenditure framework (Oranje and Mathauer 2024) and empowering district councils to draw from it. This approach could reduce reliance on fragmented project grants.

Only about 6% of surveyed residents reported getting weather or climate forecasts from the TMA for health-related climate services. This is despite the TMA’s routine issuance of quarterly malaria outlook bulletins, seasonal rainfall forecasts, and agro-meteorological updates intended to support planning in health and agriculture sectors. The low utilization highlights a critical gap in awareness, last-mile dissemination, and integration of these climate services into local-level health decision-making processes, particularly in vulnerable communities such as those in the Kilombero Valley. To respond to this gap, TMA and other partners may consider co-production of localized advisories and regularly deliver these through trusted channels such as television and radio, which are also the most common information sources in Tanzania (Table 3). To reach communities with direct key messages, engage youth and community health workers. This could improve early action as well as postimpact responsiveness of communities to climatic effects and health system disruptions.

Furthermore, data systems for early warning, such as Malaria Early Warning System can be strengthened to integrate climate and environment data to improve accuracy of predicting future threats (Ariom et al. 2022, Teklu et al. 2023). Regarding community-centred risk communication, television and radio were identified as the dominant information sources; however, people continue to rely more heavily on face-to-face village meetings and informal channels, including personal observation. This means there is a need for multimodal outreach. As such, embedding climate modules in existing community-health-worker curricula and leveraging faith leaders could widen reach and correct misconceptions that hamper behaviour change. However, the feasibility of adding climate-related content to CHW roles should be carefully assessed, given their existing workload and the need for adequate training and supportive supervision to ensure effective delivery.

In this study, farmers repeatedly cited drought-tolerant seed and timely agronomic advice as priorities. Though there are not many examples, yet, scaling climate-smart agriculture through government programmes in the agriculture sector, and where possible other sectors such as education and finance, could complement health interventions (e.g. nutrition programmes, vector control, and WASH initiatives) by stabilizing food supply and reducing malnutrition-related morbidity (Ariom et al. 2022, Teklu et al. 2023). Linking social-protection cash transfers to seasonal climate forecasts would cushion vulnerable households during failed harvests.

Although gender-disaggregated analysis was not our primary focus, there were clear gender related considerations from this study. The FGDs revealed that women carried disproportionate burdens, fetching water during drought, lost farm income, increased caregiving when children fall ill, and reduced dietary diversity during pregnancy. This echoes Pan-African evidence that female-headed households are less able to absorb climate and health-related shocks, or even to respond to them, as recently observed in the vector control scene (Ampuriire et al. 2025). Future adaptation programmes could mainstream gender-responsive budgeting and engage youth groups more effectively and meaningfully.

This study offers a baseline assessment of climate-health preparedness in Tanzania by bringing together national policy analysis, institutional perspectives, and community-level evidence. It demonstrates how gaps in coordination, financing, and information flow limit the translation of national commitments into effective local adaptation, particularly in underserved settings. By combining multiple data sources, the study highlights key implementation bottlenecks alongside priority areas for action, including climate-smart agriculture and risk communication. Overall, the findings provide actionable evidence to support more coherent multilevel governance, sustainable financing, and community-responsive planning for climate-resilient health systems.

## Study limitation

This study employed a convergent mixed-methods, multilevel assessment of climate-health preparedness in Tanzania, combining policy document review, key informant interviews, household surveys, and focus group discussions. A key strength of our approach was the convergent parallel design that triangulated policy analysis, stakeholder perspectives and household experiences in local communities. This approach enhances internal validity as the diverse informants (ministries, NGOs, researchers) enriched the analysis, while also providing opportunity for diverse stakeholder teams to participate. Nevertheless, several limitations should be acknowledged. First, the community-level surveys were conducted in a single region, the Kilombero Valley, limiting the generalisability of findings to other eco-climatic zones, such as the arid and semi-arid areas of central Tanzania. Second, this study did not include stakeholders at the regional level (e.g. Council Health Management Teams or sector leads), which represents a ‘missing middle’ in the data; therefore, the suggested actions and recommendations pertaining to this governance level have not been validated with actors operating at that level. Third, the study relied partly on self-reported data, which can be susceptible to major biases such as recall bias (Althubaiti 2016) and social-desirability bias (Krumpal 2013).

## Conclusion

This multilevel analysis demonstrates that although Tanzania has established a strong foundation of climate-health policies, significant gaps remain in translating these frameworks into effective action for frontline communities. This study provides integrated evidence showing that national preparedness and climate-related services do not yet fully match the realities faced by vulnerable households. Strengthening climate-health governance, vertical coordination from government to community level as well as risk communication strategies, will require improved coordination across sectors, sustainable and dedicated financing for adaptation, and systematic inclusion of community knowledge to design solutions that fit local contexts. A need to empower local communities with more relevant and real time data and information is also critical to ensure preparedness. Attention to gendered vulnerabilities is essential, as women disproportionately shoulder the health and livelihood burdens associated with climate variability.

Looking ahead, three priority areas emerge for future research and implementation. First, Tanzania should work towards integrating short-term and seasonal climate forecasts into routine district health planning and logistics. Second, there is a need for sustained monitoring of how combined climate shocks affect nutrition, mental health and disease patterns over time. Third, testing and evaluating innovative financing approaches such as climate-informed grants for primary health facilities will be essential for building long-term resilience. Together, these steps can help Tanzania move from policy commitment to meaningful, community-centred climate-health preparedness.

## Supplementary Material

czag066_Supplementary_Data

## Data Availability

Data collected and analysed for this study are available upon request from the corresponding author.

## Reflexivity statement

The study team comprised researchers affiliated with Tanzanian research institutions and European academic institutions. The lead author is based in Tanzania and is professionally engaged in health systems and climate-health research, which have shaped interpretation through familiarity with national policy processes and local community contexts. To minimize undue influence, data collection used standardized guides for interviews and focus group discussions, and findings were triangulated across multiple data sources (policy review, stakeholder mapping, survey, KIIs, and FGDs). Analytic interpretations were discussed among experts from different disciplinary backgrounds to challenge assumptions and improve credibility.
