# Supplementary material for: Climate-related health threats in Tanzania: a multilevel analysis of key policies, strategies, and community response
Source: Health Policy Plan. 2026 May 14;41(7):1126–39. doi: 10.1093/heapol/czag066 (PMC13481694; doi:10.1093/heapol/czag066)
Supplement: Supplementary file 1 [file czag066_supplementary_data.zip › 3.1_Rev_TABLE_1_Aloyce_Urassa.docx]

**TABLES**

Table 01: Demographic Information for survey participants from Ulanga, Malinyi, Mlimba and Ifakara town , Tanzania (N=388)

| Category | Variable | Number of participants (%) |
| --- | --- | --- |
| Sex | Male | 220 (56.7) |
|  | Female | 168(43.3) |
| Age group | 18-35 years | 147 (37.89) |
|  | 36-59 years | 187 (48.2) |
|  | 60 and above | 54 (13.92) |
|  | Mean age = 42.64 SD=13.51 |  |
| Education Level | No formal education | 15 (3.87) |
|  | Primary School | 255 (65.72) |
|  | Secondary school | 103 (26.55) |
|  | Diploma/Certificate (≥1 yr post-secondary) | 10 (2.58 |
|  | University degree (≥3 years post-secondary) | 5 (1.29) |
| Type of work or employment (main activity) | Agriculture/Farming | 246(63.4) |
|  | Entrepreneurship/Business | 117 (30.15) |
|  | Teaching | 10 (2.58) |
|  | Drivers | 05 (1.29) |
|  | Homecare | 07 (1.80) |
|  | Pastors | 03 (0.08) |
| Location | Ifakara town | 177 (45.62) |
|  | Ulanga district council | 130 (33.51) |
|  | Mlimba district council | 41 (10.57) |
|  | Malinyi district council | 40 (10.31) |
