# Supplementary material for: Climate-related health threats in Tanzania: a multilevel analysis of key policies, strategies, and community response
Source: Health Policy Plan. 2026 May 14;41(7):1126–39. doi: 10.1093/heapol/czag066 (PMC13481694; doi:10.1093/heapol/czag066)
Supplement: Supplementary file 1 [file czag066_supplementary_data.zip › 3.2_Added_Table_02_FGD_participant_characteristics.docx]

*Table 02: Characteristics of the Focus Group Discussions Participants from Ifakara town, Ulanga, Malinyi and Mlimba communities in Morogoro, Tanzania (N=64)*

| Category | Variable | n (%) |
| --- | --- | --- |
| Sex | Male | 32 (50.0%) |
|  | Female | 32 (50.0%) |
| Age (years) | Mean ± SD | 41.1 ± 11.7 |
|  | Range | 21–77 |
| Age group | 18–35 years | 24 (37.5%) |
|  | 36–59 years | 35 (54.7%) |
|  | ≥60 years | 5 (7.8%) |
| District/Council | Ifakara Mjini | 16 (25.0%) |
|  | Ulanga | 16 (25.0%) |
|  | Mlimba | 16 (25.0%) |
|  | Malinyi | 16 (25.0%) |
| Main economic activity | Agriculture | 47 (73.4%) |
|  | Entrepreneurship | 10 (15.6%) |
|  | Teaching | 5 (7.8%) |
|  | Entrepreneurship/Business | 2 (3.1%) |
| Time lived in the area (years) | Mean ± SD | 28.9 ± 16.6 |
|  | Range | 3-65 |
