# Supplementary material for: Climate-related health threats in Tanzania: a multilevel analysis of key policies, strategies, and community response
Source: Health Policy Plan. 2026 May 14;41(7):1126–39. doi: 10.1093/heapol/czag066 (PMC13481694; doi:10.1093/heapol/czag066)
Supplement: Supplementary file 1 [file czag066_supplementary_data.zip › 3.3_Rev_TABLE_3_Aloyce_Urassa_.docx]

Table 03: Common sources of information concerning climate change reported by survey participants in Ulanga, Mlimba, Malinyi and Ifakara town, Tanzania

| Source | Frequency | Percentage % |
| --- | --- | --- |
| Television | 234 | 60.3 |
| Radio | 156 | 40.2 |
| Family / friends | 51 | 13.1 |
| Local government meetings | 28 | 7.2 |
| Tanzania Meteorological Authority | 23 | 5.9 |
| Magazines | 3 | 0.8 |
| Others (mobile phones, social‑media platforms and community announcements, books, religious teachings) | 74 | 19.1 |
