# Supplementary material for: Climate-related health threats in Tanzania: a multilevel analysis of key policies, strategies, and community response
Source: Health Policy Plan. 2026 May 14;41(7):1126–39. doi: 10.1093/heapol/czag066 (PMC13481694; doi:10.1093/heapol/czag066)
Supplement: Supplementary file 1 [file czag066_supplementary_data.zip › 3.4_Rev__TABLE_4_Aloyce_Urassa_.docx]

Table 04: Climate Related Disasters faced by participants of questionnaire survey fromUlanga, Mlimba, Malinyi, and Ifakara town, in Morogoro, Tanzania

| Climate-linked hazards | Frequency (number of respondents) | Percentage (%) |
| --- | --- | --- |
| Drought | 253 | 65.2 |
| Delayed Rainfall | 211 | 54.4 |
| Extreme heat | 185 | 47.7 |
| Others (crop failure, diseases outbreak, hunger, etc.) | 154 | 39.7 |
| Floods | 121 | 31.2 |
| Unpredicted rainfalls | 120 | 30.9 |
| Extreme rainfall | 66 | 17.0 |
| Storms | 35 | 9.0 |
| Wildfires | 2 | 0.5 |
