# Supplementary material for: Climate-related health threats in Tanzania: a multilevel analysis of key policies, strategies, and community response
Source: Health Policy Plan. 2026 May 14;41(7):1126–39. doi: 10.1093/heapol/czag066 (PMC13481694; doi:10.1093/heapol/czag066)
Supplement: Supplementary file 1 [file czag066_supplementary_data.zip › 3.5_TABLE_5_Aloyce_Urassa_.docx]

Table 05: Selected survey indicators related to perceived roles and preparedness (N=388)

| Indicator (survey) | Key response options | Result |
| --- | --- | --- |
| Who should take the greatest role? (multiple responses) | Government; Community members | 71.1%; 66.0% |
| Communities can play a role to reduce health impacts | Yes | 83.5% |
| Government provides any support to help communities prepare | Yes; No; Don’t know | 73.7%; 19.6%; 6.7% |
| It is possible to control harms of climate change | Yes; No; Don’t know | 75.0%; 18.8%; 6.2% |
