# Supplementary material for: Climate-related health threats in Tanzania: a multilevel analysis of key policies, strategies, and community response
Source: Health Policy Plan. 2026 May 14;41(7):1126–39. doi: 10.1093/heapol/czag066 (PMC13481694; doi:10.1093/heapol/czag066)
Supplement: Supplementary file 1 [file czag066_supplementary_data.zip › SUPPLEMENTARY_MATERIALS_2026_AloyceUrassa.docx]

**SUPPLEMENTARY MATERIALS**

Title: **Climate-related health threats in Tanzania: a multi-level analysis of key policies, strategies and community response**

**Table S1:** Timeline of Tanzania’s actions and policies relevant to addressing health threats due to climate change

| Date | Climate-health milestone | References |
| --- | --- | --- |
| Jun 1992 | Tanzania signed the UN Framework Convention on Climate Change (UNFCCC). | United Nations. United Nations Framework Convention on Climate Change: Status of Ratification, Tanzania [Internet]. New York: UN; 1992 [cited 2025 Nov 28]. Available from: <https://treaties.un.org/Pages/ViewDetailsIII.aspx?src=IND&mtdsg_no=XXVII-7&chapter=27&Temp=mtdsg3&clang=_en> |
| Apr 1996 | Parliament ratified the UNFCCC, formalizing national obligations. | United Nations Treaty Collection. UNFCCC United Republic of Tanzania Ratification Details [Internet]. New York: UN; 1996 [cited 2025 Jan 7]. Available from: <https://treaties.un.org/Pages/ViewDetailsIII.aspx?src=IND&mtdsg_no=XXVII-7&chapter=27&Temp=mtdsg3&clang=_en> |
| Aug 2002 | Government ratified the Kyoto Protocol, committing to emission cuts. | United Nations Treaty Collection. Kyoto Protocol – United Republic of Tanzania Ratification [Internet]. New York: UN; 2002 [cited 2025 Nov 28] Available from: <https://treaties.un.org/pages/ViewDetails.aspx?src=IND&mtdsg_no=XXVII-7-a&chapter=27> |
| Jan 2006 | National Adaptation Programme of Action (NAPA) was issued; human health ranked among 14 urgent priorities. | Vice President’s Office. National Adaptation Programme of Action (NAPA) for Tanzania [Internet]. Dar es Salaam: VPO; 2007 [cited 2025 Nov 28]. Available from <https://unfccc.int/resource/docs/napa/tza01.pdf> |
| Aug 2009 | Tanzania endorsed the Libreville Declaration on Health & Environment in Africa. | WHO Regional Office for Africa. Libreville Declaration on Health and Environment in Africa: Commitments by Member States [Internet]. Brazzaville: WHO AFRO; 2009 [cited 2025 Nov 28]. Available from: <https://www.afro.who.int/publications/libreville-declaration> |
| Jan 2012 | Stakeholders validated the draft National Climate Change Strategy (NCCS), which embedded health objectives. | Vice President’s Office. National Climate Change Strategy (NCCS) 2012 [Internet]. Dar es Salaam: VPO; 2012 [cited 2025 Nov 28]. Available from: <https://climhealthafrica.org/wpostahs-slider-category/tanzania/#:~:text=14%20Sep%20January%202012&text=A%20national%20stakeholder%20workshop%20is,capacities%20within%20social%20facilities>... |
| Feb 2014 | Global Framework for Climate Services (GFCS) Adaptation for Africa programme was launched; a project-delivery team on climate & health was formed. | World Meteorological Organization. GFCS Adaptation Programme in Africa: Tanzania Launch Report [Internet]. Geneva: WMO; 2014 [cited 2025 Nov 28]. Available from: [https://wmo.int/activities/projects/project-portfolio/climate-services-adaptation-programme-africa-malawi-tanzania#:~:text=A%20national%20consultation%20embracing%20all,of%20the%20three%2Dyear%20period](https://wmo.int/activities/projects/project-portfolio/climate-services-adaptation-programme-africa-malawi-tanzania#:~:text=A%20national%20consultation%20embracing%20all,of%20the%20three%2Dyear%20period). |
| May 2014 | A national workshop mapped institutional gaps in climate services for health, agriculture and Disaster Risk Reduction. | World Meteorological Organization. Tanzania National Consultation Workshop on Climate Services Report [Internet]. Geneva: WMO; 2014 [cited 2025 Nov 28]. Available from: [https://wmo.int/activities/projects/project-portfolio/climate-services-adaptation-programme-africa-malawi-tanzania#:~:text=A%20national%20consultation%20embracing%20all,of%20the%20three%2Dyear%20period](https://wmo.int/activities/projects/project-portfolio/climate-services-adaptation-programme-africa-malawi-tanzania#:~:text=A%20national%20consultation%20embracing%20all,of%20the%20three%2Dyear%20period) |
| Jan 2016 | Tanzania government published the National Climate Change and Health Communication Strategy. | Ministry of Health. National Climate Change and Health Communication Strategy 2016–2021 [Internet]. Dar es Salaam: MoH; 2016 [cited 2025 Nov 28]  Available from: <https://climhealthafrica.org/news-tanzania-develops-national-climate-change-and-health-communication-strategy/> |
| Apr 2016 | Tanzania signed the Paris Agreement, reaffirming mitigation and adaptation commitments. | United Nations. Paris Agreement – Status of Ratification: Tanzania [Internet]. New York: UN; 2016 [cited 2025 Nov 28]. Available from: <https://treaties.un.org/pages/ViewDetails.aspx?src=TREATY&mtdsg_no=XXVII-7-d&chapter=27> |
| Mar 2018 | Health National Adaptation Plan (HNAP) 2018–2023 was finalized to guide a climate-resilient health system. | Ministry of Health. Health National Adaptation Plan (HNAP) 2018–2023 [Internet]. Dar es Salaam: MoH; 2018 [cited 2025 Nov 28]. Available from: <https://www.atachcommunity.com/fileadmin/uploads/atach/Documents/Country_documents/Tanzania_HNAP_2018.pdf> |
| Nov 2023 | Tanzania endorsed the COP28 Climate and Health Declaration at United Arab Emirates | COP28 Declaration and Climate and Health;  <https://cdn.who.int/media/docs/default-source/climate-change/cop28/cop28-uae-climate-and-health-declaration.pdf> |
| 2024 – 2025 | The HNAP 2024–2030 revision and third Nationally Determined Contribution (NDC 3) review were initiated. |  |

Table **S2:** Stakeholder Categorisation Framework for Climate-Health Preparedness in Tanzania

| Level | Stakeholder Group | Role | Influence/Engagement Level |
| --- | --- | --- | --- |
| Individual | Household members, youth, women, farmers, Community Health Workers (CHWs) | Directly experience climate-health impacts; agents of behavioural change | Low |
| Community | Local CHWs coordinators, village leaders, school teachers | Local mobilisation, awareness campaigns, linking health, education & climate literacy | Low |
| Community | Local civil society organisations and youth led organizations | Community-level implementation of adaptation projects, education, and early warning | Medium |
| Regional | District Health Management Teams (CHMTs)  District Environmental Officers, District Agricultural Officers | Lead planning and implementation of health adaptation and resilience plans  Translate national policies into district-specific action; monitor ecosystem-health links | High |
| Regional | Regional Health Management Teams (RHMTs), regional level NGOs | Implementation of policies at regional level | Medium |
| National | Ministry of Health (MoH) | Policy leadership, HNAP coordination, integration into health system planning | High |
| National | Tanzania Meteorological Authority (TMA) | Provides climate forecasts, malaria outlooks, early warning services | High |
| National | Prime Minister’s Office-Regional Administration and Local Government (PMO-RALG) | Oversight of local government health and development integration | High |
| National | Vice President’s Office (Environment) | Coordinates climate policy, Nationally Determined Contributions (NDCs), and sectoral mainstreaming of climate risks | High |
| National | Ministry of Finance | Budget allocation, Medium-Term Expenditure Framework (MTEF), climate financing | High |
| National | Parliamentarians, Parliamentary Committees on Health or Environment | Legislative oversight and resource advocacy | High |
| National | Researchers, national NGOs and Civil Society Organizations (CSOs), and academic institutions | Evidence generation, policy advice, evaluation of interventions, climate and health project designing and implementation , advocacy | Medium |
| International | Donors and development partners (e.g. WHO, Green Climate Fund, Gavi, World Bank) | Provide technical assistance and funding for climate-health projects | High |
